# L-lactate as an indicator for cellular metabolic status: An easy and cost-effective colorimetric L-lactate assay

**DOI:** 10.1371/journal.pone.0271818

**Published:** 2022-07-22

**Authors:** Kira Schmiedeknecht, Andreas Kaufmann, Stefan Bauer, Francisco Venegas Solis

**Affiliations:** Institute for Immunology, Philipps-University Marburg, BMFZ, Marburg, Germany; Southern Illinois University School of Medicine, UNITED STATES

## Abstract

**Background:**

In recent times, the study of metabolic pathways has become inevitable and predominant for a variety of research fields as cancer biology and immunology. L-lactate as a product of anaerobic glycolysis has shown to be an important indicator of the cellular metabolic status and can be associated with diverse cellular effects. For this reason, L-lactate assay kits are of high demand when metabolic effects need to be considered. Nevertheless, commercially available kits are not affordable if multiple samples must be evaluated.

**Principal finding:**

In this work, we develop an easy and cost-effective colorimetric assay for quantification of L-lactate suitable for cells with low or high L-lactate production based on LDH activity and suitable for 96 well-plate format. Using different metabolic regulators, we demonstrate the capacity of the assay to detect and quantify L-lactate from the supernatant of HeLa cancer cell line. Furthermore, we validate the assay against a commercially available kit by demonstrating no significant difference between both assays. Finally, we show that the assay is capable of quantifying L-lactate in primary cells such as hPBMCs that were stimulated with toll-like receptor ligands and treated with different metabolic regulators.

**Conclusion:**

We herein present an easy custom assay that is suitable for cells with low and high L-lactate production at very low cost compared to commercially available kits. These advantages of the custom assay can simplify the research in the field of metabolism and related fields.

## Introduction

When it comes to fulfilling their energy needs, cells can utilize different metabolic pathways to achieve that goal. For these processes, nutrients are delivered to and consumed by cells to provide adenosine triphosphate (ATP) as a source of energy. Generated ATP allows for basic yet profound cellular functions as DNA repair, transcription and translation, formation of biological building material and ultimately cellular homeostasis to occur [1]. As glycolysis is a process being available for all eukaryotic cells, it plays a superior role in the metabolism of the cells in the human body.

Firstly in the glycolytic pathway, glucose is metabolized into two molecules of pyruvate with a net production of two ATP molecules [2]. In the following, dependent on the oxygen supply of the cells either an aerobic or an anaerobic pathway can be differentiated. While the aerobic pathway involves a transformation of pyruvate to acetyl-CoA, which ultimately results in a production of high ATP amounts through oxidative phosphorylation (OXPHOS), the anaerobic glycolysis reduces pyruvate to L-lactate by the enzyme L-lactate dehydrogenase (LDH) to regenerate nicotinamide adenine dinucleotide (NAD^+^). Accordingly, the amount of L-lactate indicates the metabolic status of cells in general.

There exist two enantiomers of lactate: D-lactate and L-lactate the latter being the predominant physiological enantiomer present in humans [3, 4].

While under normoxic conditions cells are able to utilize aerobic metabolism to gain higher amounts of ATP, some cells nevertheless prefer anaerobic glycolysis even when enough oxygen is supplied. This is a characteristic feature of cancer cells called the Warburg effect [5]. Since the background of why this reprogramming towards anaerobic glycolysis is occurring especially regarding the inefficiency for energy outcome compared to an aerobic approach and which consequences on cellular function it may have is still not fully understood [1, 6]. Cancer cells, however, are not the only focus of interest when metabolism is investigated. In recent years, growing knowledge of the metabolic activity of immune cells enhanced curiosity in cellular regulations and functions [7–9]. Conclusively, the diverse attention drawn to metabolic investigation needs to be met with accessible experimental methods.

L-Lactate is an important indicator of the metabolic status of cells and due to this, determination of its concentration in cells becomes important in the study of metabolism and its implications. Commercially available kits determine L-lactate concentration in sample supernatants and thereby might be helpful for research in the abovementioned fields. Nevertheless, these kits are expensive when multiple samples need to be measured as for biological or experimental replicates. Additionally, the accuracy is only given for a very small range at low L-lactate concentrations resulting in difficulties for L-lactate measurements in cell lines with higher glycolytic rate (e.g. cancer cell lines) since samples would need to be diluted 1:20 or more for accurate results.

The assay for L-lactate measurement we present hereafter protrudes by its simplicity and cost-effectiveness. We show precise and reproducible results for a wide range of L-lactate concentrations. An established colorimetric reaction was employed for optimal accuracy. Our L-lactate assay measures L-lactate concentrations in cancer and primary cells (hPBMCs) and supports the analysis of cellular treatment with metabolic regulators. The assay approach enables important research by allowing access to detection of L-lactate concentrations and thus determination of cellular metabolic mode.

## Material and methods

### Reagents

Iodonitrotetrazolium chloride (INT), β-nicotinamide adenine dinucleotide (NAD), L-lactate dehydrogenase (LDH), sodium L-lactate and the commercial L-lactate assay kit were acquired from Sigma-Aldrich Company; methoxy phenazine methosulfate (M-PMS) was purchased from MedChemExpress. Also, metabolic regulators as sodium dichloroacetate (DCA), 2-deoxy-D-glucose (2-DG) and 1,1-dimethylbiguanide hydrochloride (metformin) were obtained from the Sigma-Aldrich Company and subsequently dissolved in H_2_O. Lipopolysaccharide from Escherichia coli O127:B8 was purchased by Sigma-Aldrich Company and R848 delivered B8 (LPS) by InvivoGen.

### Ethics statement

The use of anonymous blood samples for this study was approved at the local ethic committee of the Justus-Liebig-University Giessen and Philipps-University Marburg. The human samples (buffy coats from blood donors) were provided by the Institute for Clinical Immunology and Transfusion Medicine, Justus-Liebig-University Giessen, Germany. We confirm that all methods for drawing blood and preparation of buffy coats were performed in accordance with local guidelines and regulations. We also confirm that blood products were obtained only after informed consent from the blood donors. Human peripheral blood mononuclear cells (PBMCs) were isolated from buffy coats by Ficoll density gradient centrifugation with LSM 1077 (PAA).

### Cell culture

HeLa Cells were cultured in RPMI medium supplemented with 10% fetal calf serum (FCS), 1% penicillin and streptomycin in a 5% CO_2_ humidified atmosphere at 37°C.

### Treatment of HeLa cells with different metabolic regulators

HeLa cells were cultured (150 μL of 10x10^6^ live cells/mL) in a 96-well plate. They were treated with three different concentration of 2-DG (320, 160 and 80 mmol/L), DCA (40, 20 and 10 mmol/L), metformin (20, 10 and 5 mmol/L) or RPMI medium as a negative control and were incubated for 24 h, 48 h and 72 h at 37°C, 5% CO_2_. After the incubation, the culture supernatants were collected and pH and L-lactate concentration were determined by the modified pH method and the L-lactate assay. The conditions tested were analyzed by duplicates in every individual experiment.

### Treatment of hPBMC with different glycolysis inhibitors

Human PBMCs were cultured (100 μL of 3x10^6^ live cells/mL) in a 96-well plate. Cells were treated with two different concentration of 2-DG (160 and 80 mmol/L), DCA (40 and 10 mmol/L), metformin (20 and 10 mmol/L) or RPMI medium as a negative control and were incubated for 1 h at 37°C, 5% CO_2_. Then cells were treated with R848 (1 μmol/L) or LPS (10 ng/mL). After incubating for 20 h, the culture supernatants were collected and used to measure the concentration of IL-6 by ELISA according to the manufacturer’s instructions as well as the L-lactate concentration by the L-lactate assay.

### Cytokine measurement

Concentration of IL-6 human in the culture supernatant were measured by ELISA according to the manufacturer’s instructions (Pharmingen). Cytokine production significantly above untreated control was interpreted as an activation of gene expression.

### pH measurement

pH measurement was assessed using the absorbance of phenol red (PhR) according to Michl J. [10], with modifications. In brief, 10 μL of PhR (2,8 mmol/L) were added to RPMI medium prepared with different amounts of 2 mol/L HCl or 2 mol/L NaOH to a total volume of 500 μL in order to generate different pH values. pH was first determined with a pH meter followed by absorbance measurement (430 and 560 nm) with an absorbance reader (Nanodrop). Lastly, the measured absorbance was used to establish a standard curve with the pH values determined with the pH meter and the calculated equation was utilized for pH determination of later samples. For measurement of the samples, 1 μL of PhR (2,8 mmol/L) was added to 49 μL of sample supernatant, absorbance was directly ascertained and pH determined as described above. The addition of PhR to the medium facilitated the measurement of the absorbance and the change it undergoes at different pH.

### L-Lactate assay

After the treatment, 50 μL of the supernatant of the different conditions or a dilution of these were used for the quantification of the amount of L-lactate.

50 μL of either L-lactate standard or supernatant of samples (diluted or undiluted) was added to a well of a 96-well plate followed by addition of 50 μL of premixed reaction buffer. The reaction buffer of a total volume of 5 mL was prepared with tris-base (170 mmol/L), β-NAD (17 μmol/L), INT (4,9 μmol/L), L-LDH (6,9 μmol/L) and M-PMS (0,25 μmol/L). L-Lactate standards with the concentrations of 12–0,375 mmol/L were prepared from sodium L-lactate in RPMI medium. Reactions were incubated for 1 h at room temperature and in dark before they were stopped by addition of 50 μL acetic acid (1 mol/L). Lastly, absorbance was recorded with a microplate reader at 490 nm—650 nm (reference) and data was normalized to only RPMI treated like abovementioned. The standard curve was used for calculating the concentration of L-lactate present in the well of the 96-well plate.

The following stock solutions were used for the L-lactate assay: INT (19,8 mmol/L; in 67% MeOH), β-NAD (33,2 mmol/L), L-LDH (34,7 mmol/L), M-PMS (74,3 mmol/L). Stock solutions were aliquoted, stored at -20°C and thawn for preparation of the reaction buffer the day they were used.

### hPBMC and L-lactate assay

For measurement in cells incubated with RPMI medium containing sodium pyruvate, enzyme concentration needs to be adjusted to 41,4 μmol/L of L-LDH, as we experienced enzyme inhibition by pyruvate for low concentrations of L-LDH in this condition.

In addition, the absorbance recorded with the microplate reader has to be set to 490 nm without a reference of 650 nm. The remaining steps should be followed according to the description above.

### L-Lactate assay kit (Sigma-Aldrich)

To validate our protocol, our measurements were compared to the measurement with a commercially available L-lactate assay kit (MAK329-1KT, Sigma-Aldrich, Darmstadt, Germany). L-Lactate standards were measured according to the manufacturer’s instructions to evaluate accuracy.

### Statistical analysis

Statistical significance of the effect of different metabolic regulators in cytokine, L-lactate production and acidification in HeLa cells or hPBMC was determined by analysis of variance (ANOVA). A significant effect in ANOVA was analyzed using Dunnett’s post-hoc test and the control was compared against the conditions. P value lower than 0,05 was considered statistically significant.

In the case of analysis of linear regression, the slope, limit of detection and the coefficient of determination were used as criteria for best fitting curve.

## Results

### Establishment of the L-lactate assay conditions

**Fig 1:**
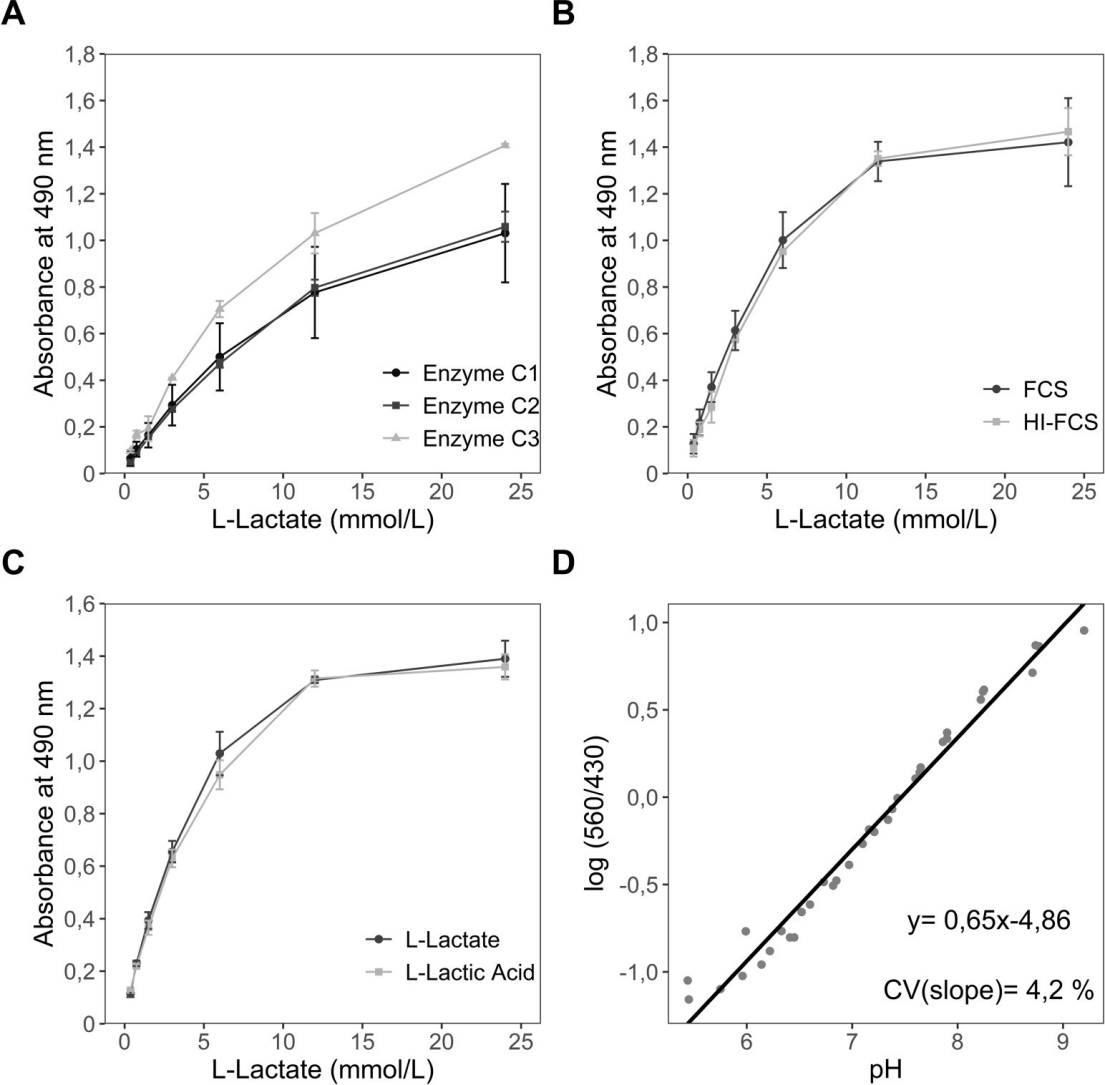
Establishing conditions for L-lactate and pH measurement. (A) Establishment of the conditions for the L-lactate measurement assay was achieved by testing of three different substrate concentrations (Iodonitrotetrazolium chloride; c = 1 mmol/L, 2 mmol/L, 3 mmol/L; only 1 mmol/L shown in graphic) and three different enzyme concentrations (L-lactate dehydrogenase; C1 = 1,73 μmol/L, C2 = 3,45 μmol/L, C3 = 6,9 μmol/L). Two different possible influencing factors (B) FCS and (C) pH on accuracy of measurement were tested. (D) Determination of pH was obtainable from PhR absorbance spectrum and was utilized for calculation of the equation being used for subsequent pH measurements. (A)-(C) The data represents the mean ± sd of two independent experiments. (D) The data represents the mean ± sd of three independent experiments. (A) Pearson´s correlation (r^2^) = 0,99. For (B) and (C) there is no significance between the conditions tested according to the results of ANOVA.

In order to find the appropriate conditions for measurement of L-lactate in the supernatant of cells using our custom L-lactate assay, we tested different concentrations of LDH and INT in the assay buffer and two time points of incubation of the reaction. Absorbance of various L-lactate standards was measured and subsequently values were used to construct L-lactate standard curves (Fig 1A, only shows three conditions of nine for simplicity). We evaluated the different curves according with sensibility (slope), lower limit of quantification, coefficient of determination and economical choice. The best model according with our established criteria presents an enzyme concentration of 6,9 μmol/L, a substrate concentration of 1 mmol/L and 1 h incubation.

As the presented assay relies on a functioning enzyme reaction, possible influential factors needed to be considered. First, it was tested whether the amount and activity of the L-lactate dehydrogenase contained by the FCS added to the RPMI cell culture medium could influence the measurement accuracy by comparison of standard measurements conducted with RPMI containing FCS and RPMI containing heat-inactivated FCS (HI-FCS) (Fig 1B). Secondly, the effect of the pH was examined by comparison of standard measurements prepared with either L-lactate or L-lactic acid (Fig 1C). Both factors were not showing significantly different values compared to negative controls (with FCS or L-lactate) and for this reason were not considered as disruptive factors for the L-lactate assay.

Since anaerobic metabolism in cells leads to accumulation of L-lactate as well as to acidification of the medium (pyruvate-> L-lactate + H^+^), we considered that another important factor to study together with L-lactate measurement is the change of the pH in the medium and the correlation of pH and L-lactate production. Fig 1D exhibits a pH standard curve constructed with PhR absorbance according to the protocol of Michl et al. [10], which is described in further detail in methods and was used to determine pH in later experiments.

### Testing the L-lactate assay in cell culture

**Fig 2:**
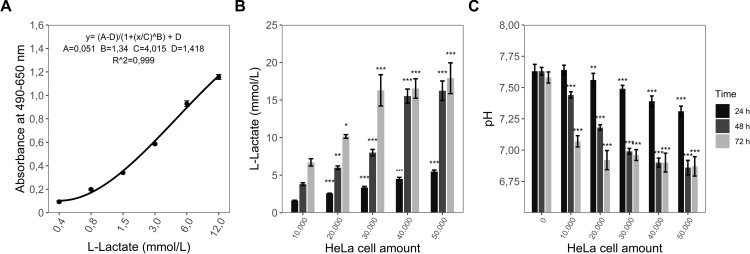
L-Lactate determination in HeLa cell culture. (A) Example of a standard curve and the equation for quantification of L-lactate in the custom assay. Different cell amounts of HeLa cells were seeded in 96-well plate format and cultured in RPMI Medium. After 24h, 48h and 72h (B) L-Lactate concentration and (C) pH were measured using the described protocols. The data represents the mean ± sd of two independent experiments. (A) *p < 0,05 compared to 10.000 cells, **p < 0,01 compared to 10.000 cells and ***p < 0,001 compared to 10.000 cells. (B) *p < 0,05 compared to RPMI Medium, **p < 0,01 compared to RPMI Medium and ***p < 0,001 compared to RPMI Medium.

For validating that the L-lactate assay can measure L-lactate production of cells grown in culture over time, HeLa cells were seeded and incubated over a total of 72 h. At three time points, 49 μL of the supernatant was used for pH measurement with PhR while other 50 μL were used for the quantification of L-lactate using a standard curve (Fig 2A) generated by the custom assay. As expected, the L-lactate assay measured significantly increasing L-lactate concentrations in the supernatant depending on initial cell concentration and incubation time. However, at high cell density L-lactate production reached a maximum (16,24 ± 1,30 mmol/L) already at 48h incubation time (Fig 2B). Simultaneously, pH significantly decreases for all cell amounts and all incubation times (except 10.000 cells over 24 h) compared to medium control (Fig 2C). The results show the ability of the L-lactate assay to recognize L-lactate production in cell culture. In addition, our measurements show that L-lactate release of HeLa cells correlates with a decrease in pH for 24 h and 48 h (Pearson´s r = -0,99 (24 h) and r = -0,97 (48 h)).

### Metabolic regulators and the influence on L-lactate production and pH

**Fig 3:**
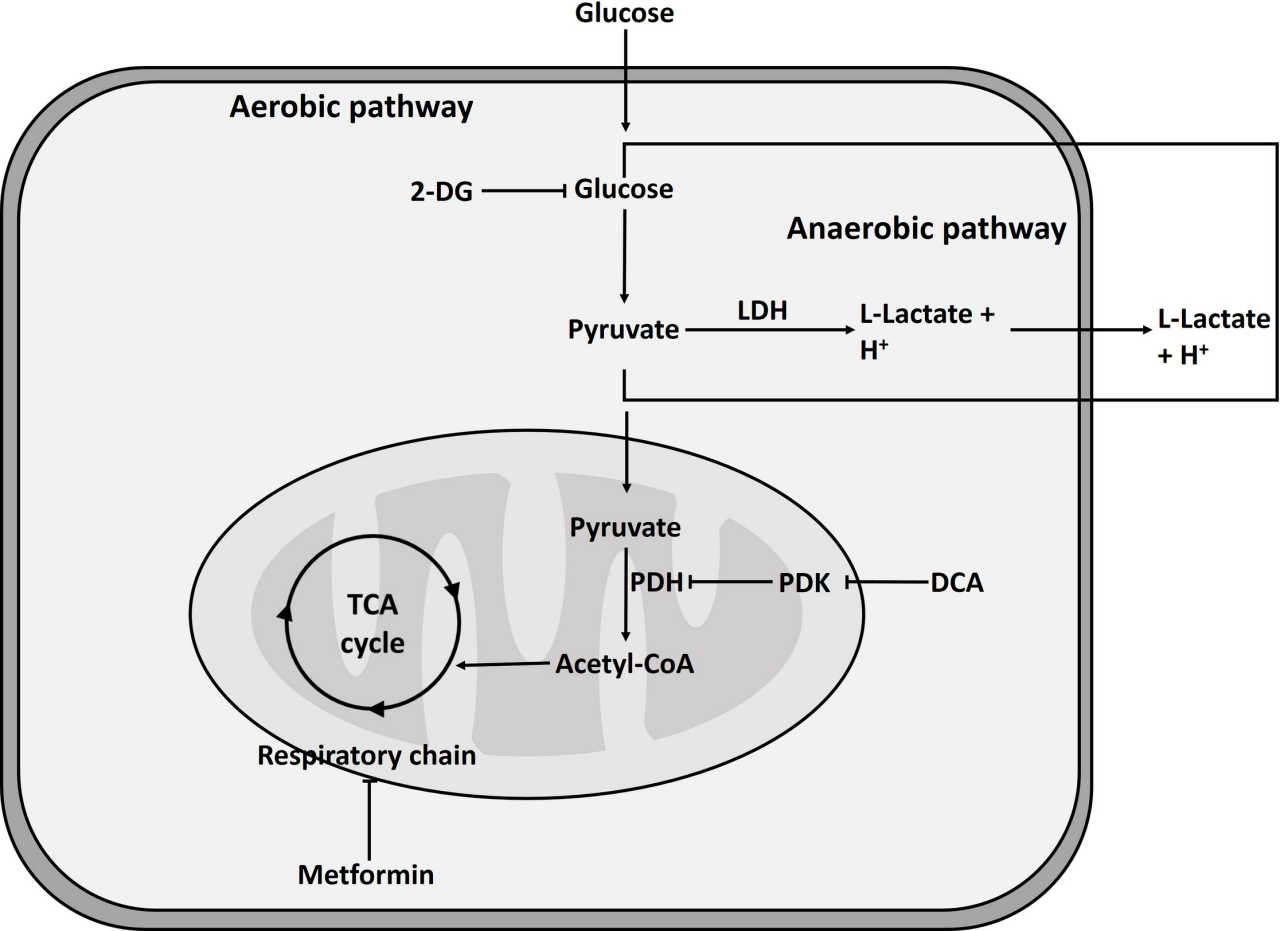
Regulation of aerobic and anaerobic pathway by several metabolic regulators. Schematic representation of utilized glycolysis regulators and their targets in the aerobic and anaerobic pathway. LDH = lactate dehydrogenase; PDH = pyruvate dehydrogenase; PDK = pyruvate dehydrogenase kinase; TCA cycle = tricarboxylic acid cycle.

Most cells fulfill their energy needs by oxidative phosphorylation as this pathway allows for a higher ATP generation. Under hypoxic conditions, cells need to reprogram their metabolism towards anaerobic glycolysis to gain energy. Certain metabolic regulators as dichloroacetate (DCA) and 2-deoxyglucose (2-DG) reduce this reprogramming and therefore L-lactate production decreases. In contrast, metformin as an inhibitor of complex II of the respiratory chain increases L-lactate production [7] (Fig 3).

**Fig 4:**
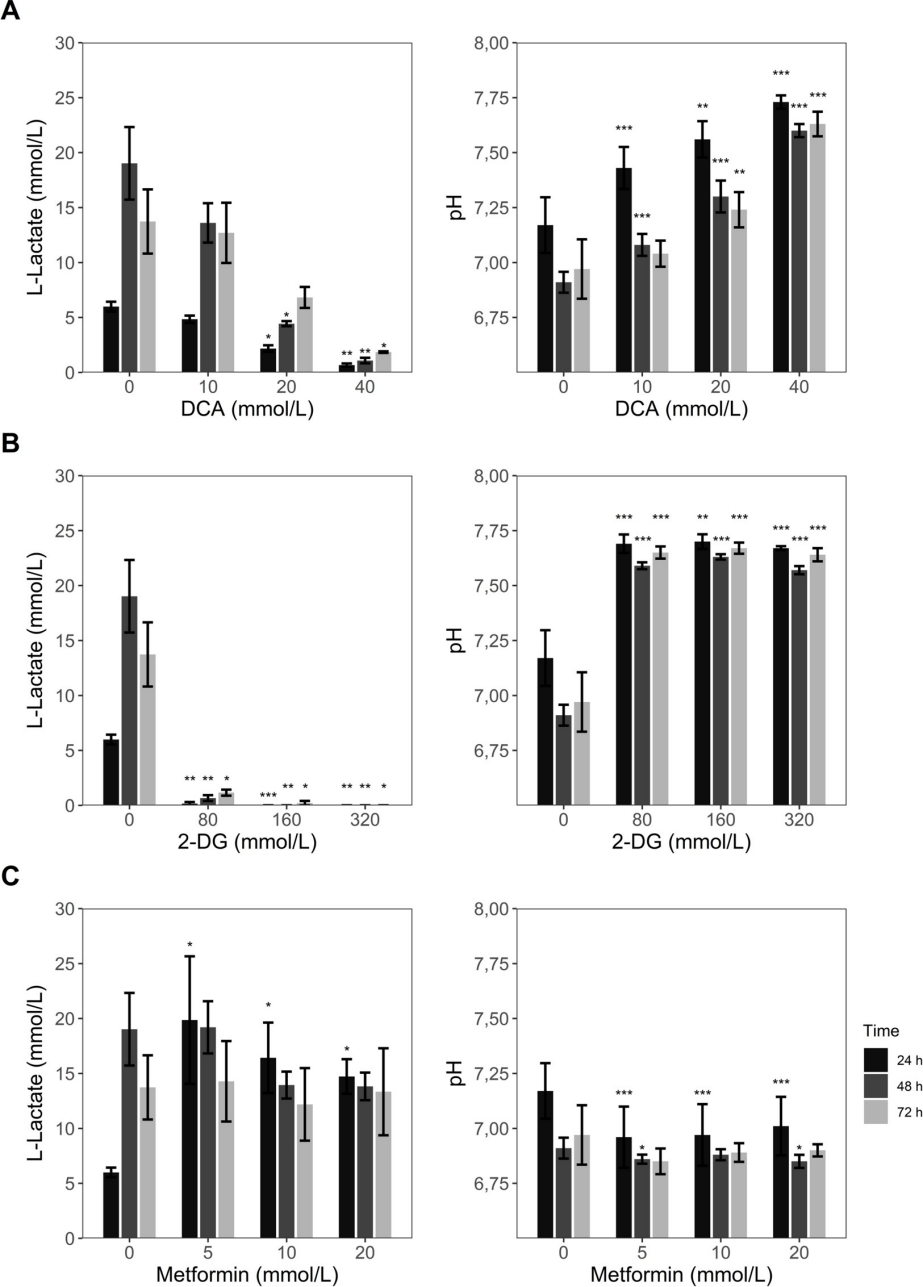
L-Lactate determination upon treatment with metabolic regulators. HeLa cells (50.000 cells/well) were plated in 96-well plate format with direct addition of the described metabolic regulators. After 24 h, 48 h and 72 h L-lactate concentration and pH were determined for (A) DCA, (B) 2-DG and (C) metformin conditions. The data represents the mean ± sd of two independent experiments. *p < 0,05 compared to untreated control, **p < 0,01 compared to untreated control and ***p < 0,001 compared to untreated control.

The influence of these inhibitors on L-lactate release and pH variation was tested in HeLa cells at different time points using the L-lactate assay and PhR absorbance. Expected significant reduction of L-lactate concentration was visible for the two highest concentrations of DCA while the pH showed an increase even in the lowest concentration of DCA (Fig 4A). In the case of 2-DG (Fig 4B), the concentrations tested abolished the production of L-lactate and an increase of the pH was seen for all the conditions tested, whereas metformin treatment enhanced L-lactate release and decreased pH significantly for all concentrations only in the first 24 h (Fig 4C). These results demonstrate that the L-lactate assay shows valid results for measurement of metabolic activity in cultured cells after treatment with metabolic regulators.

### Comparison to commercially available L-lactate assay kit

The performance of the L-lactate assay was tested against the commercially available L-lactate assay kit (Sigma-Aldrich).

**Fig 5:**
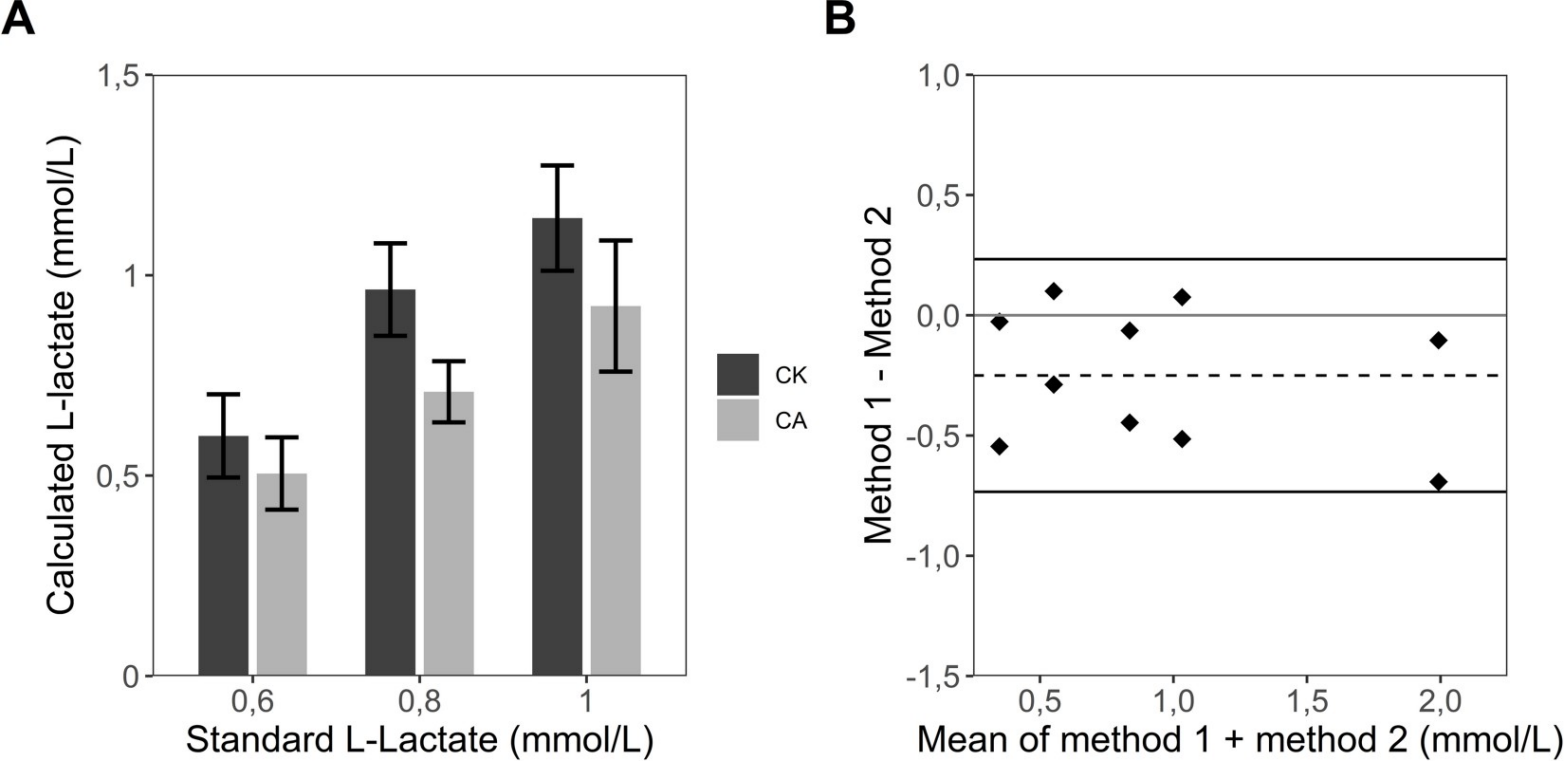
Comparison to commercially available L-lactate assay kit. Precision of L-lactate determination by the custom assay (CA) was tested against commercial kit (CK) (A). In the Bland-Altman-Plot (B) every point represents the difference between measured values of the custom L-lactate assay and those of the commercial L-lactate assay kit from one experiment and the graphic shows two independent experiments. The black lines demonstrate limits of agreement (LoA), grey line shows perfect agreement and discontinuous line exhibits the mean of the difference between the two assays. The data represents the mean ± sd of two independent experiments.

L-Lactate standards (0,4–2 mmol/L) were prepared and measured according to protocol of the commercial kit and the custom L-lactate assay. As limit values of the accuracy of the commercial kit (2 mmol/L) and the custom L-lactate assay (0,4 mmol/L) these two concentrations were eliminated from the statistical analysis. For the other standard L-lactate concentrations, there is no significant difference in values calculated from both L-lactate assays (Fig 5A). The Bland-Altman-Plot (Fig 5B) depicts that the two methods are essentially equivalent, since the differences of the various values measured by the methods (single points) spread within the limits of agreement and the mean of the differences is close to the line of perfect agreement. The graphic also depicts that the custom assay on average slightly underestimates concentration of L-lactate compared to the commercial kit being visible in our mean of differences proceeding underneath the line of perfect agreement.

While we demonstrated, that both assays are equally precise in measuring correct L-lactate concentrations, the custom assay has several advantages over the commercial L-lactate kit by Sigma-Aldrich. First, the developed method accurately measures a much wider range of L-lactate concentrations (0,375 mmol/L– 12 mmol/L) compared to the kit with its limits being 0,1 mmol/L– 2 mmol/L. The range of the latter test additionally decreases to 0,1 mmol/L– 1 mmol/L when the cellular medium contains the indicator phenol red, which is commonly present in e.g. standard RPMI or DMEM medium. However, the quite small range of L-lactate concentrations measurable with the commercial kit exhibits a problem for its validity for cell lines with high L-lactate production levels. The results for L-lactate production in HeLa cells (Fig 2) show concentrations of 5 mmol/L of L-lactate after only 24h and even 20 mmol/L of L-lactate and more over a longer time period.

Accordingly, measuring such samples with the commercial kit requires high dilutions of the cellular supernatants to fit within the limits. Due to this, the commercial kit is not suitable for research of cell lines with an increased glycolytic rate, at the same time the custom assay presented in this work shows a high performance for studying cell lines with a high production of L-lactate.

Although our results only illustrate the high glycolytic activity of HeLa cells, this cancer cell line represents the metabolic status of various types of cancer. Assumably, other cancerous cells may also exceed the L-lactate concentration limits of the commercial kit especially upon stimulation by hypoxia or cellular stress known to enhance L-lactate levels [11]. Additionally, studies of metabolic activity in commonly used non-cancerous cells like HEK-293 [12] or primary cells like hPBMCs (in following) measured L-lactate concentrations far higher than the 1–2 mmol/L limit of the Sigma-Aldrich kit. Therefore, we may assume that the developed custom assay is more suitable for several types of cultured cells with normal to high glycolytic activity.

The second major advantage of the custom assay is its cost-effectiveness compared to the commercial kit. In Table 1, prices of the different compounds of the assay required for 100 reactions are presented and compared to the pricing of the commercial kit suitable for 100 reactions. It is obvious that the custom assay is 260-fold less expensive at $ 1,47/ 100 reactions than the commercial kit at $ 381/ 100 reactions. Especially for experiments with numerous samples or treatments and of course the necessity of experimental replications, pricing is an issue to consider. As the two methods are both equally accurate, take similarly long to prepare and use the same laboratory equipment, this major economic advantage increases the attractiveness of the custom assay significantly.

**Table 1:** Cost of the reagents of custom L-lactate assay.

Reagents	Amount per 100 reactions (μL or μg)	Price per 100 reactions ($)
Acetic Acid (glacial)	290	0.02
β-Nicotinamide Adeine Dinucleotide Sodium salt (NAD)	1100	0.97
Iodonitrotetrazolium Chloride (INT)	2500	0.22
L-Lactate Dehydrogenase (L-LDH)	10	0.07
1-Methoxyphenazine methosulfate (1-Methoxy-PMS)	75	0.11
Sodium L-lactate	2252	0.05
2-Amino-2-(hydroxymethyl)-1,3-propandiol (Tris Base)	100	0.03
**Total**		**1.47**

Regarding the time of the assays, the commercial kit needs 20 min while the custom assay needs 1 h incubation time. In this case, the commercial kit is superior to the custom assay. However, the custom assay is superior in costs and concentration range that can be analyzed.

### Effect of metabolic regulators on L-lactate and IL-6 production in hPBMCs

The effect of the before mentioned metabolic regulators (Fig 3) DCA, 2-DG and metformin on L-lactate and IL-6 production in hPBMCs was investigated for validation of the L-lactate assay for primary cells.

**Fig 6:**
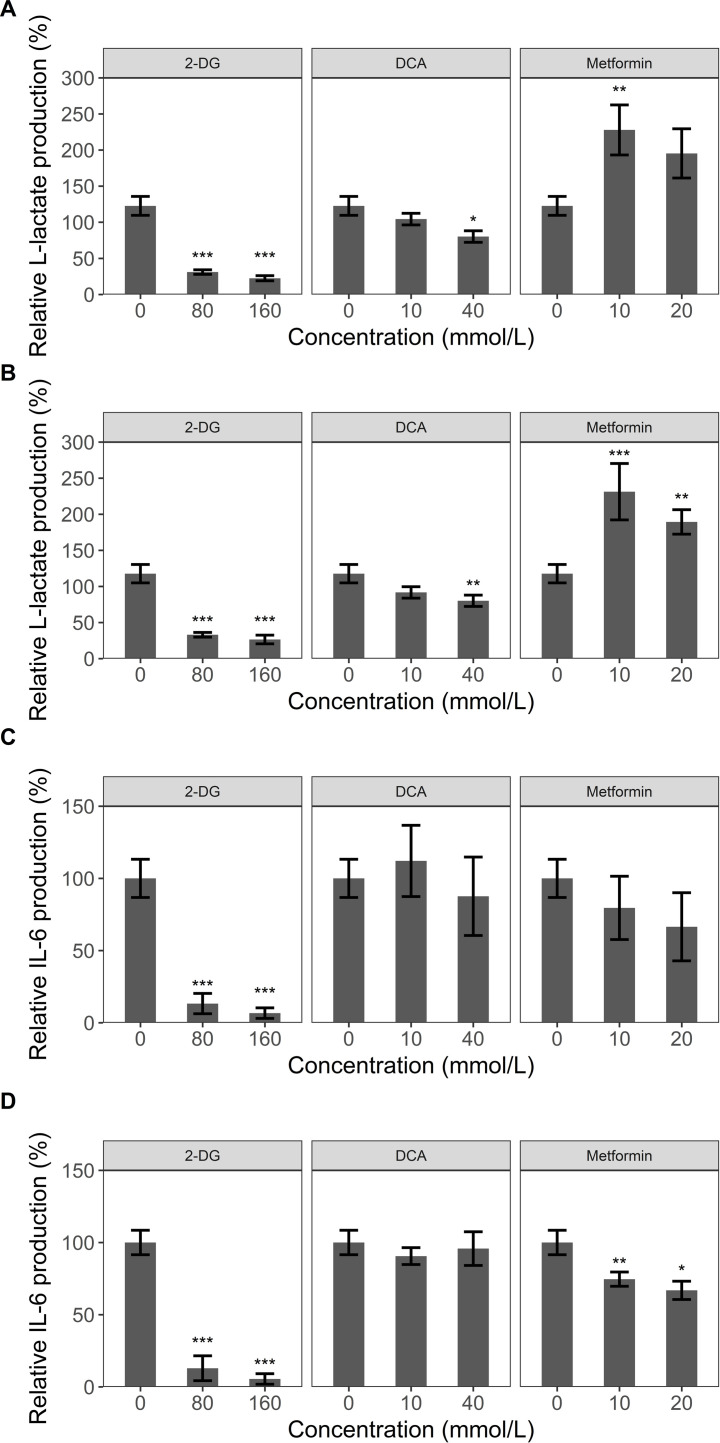
L-Lactate measurement in Co-stimulated hPBMCs. Human PBMCs were seeded and incubated with DCA, 2-DG and metformin for one hour. Then stimulation with LPS (10 ng/mL) (A)+(C) or R848 (1 μmol/L) (B)+(D) followed while control conditions were left untreated. After 20 h L-lactate production (A)+(B) and IL-6 production (C)+(D) were determined measuring the supernatant by L-lactate assay and ELISA. Relative L-lactate production was defined as the percent ratio of L-lactate in treated cells compared to the medium control. Relative IL-6 production was defined as the percent ratio of IL-6 in treated cells compared to the untreated control. The data represents the mean ± sd of four donors. *p < 0,05 compared to untreated control, **p < 0,01 compared to untreated control and ***p < 0,001 compared to untreated control.

In presence of lipopolysaccharide (LPS), higher L-lactate production (120 ± 13%) was observed compared to medium control (100%). Of note, 80 and 160 mmol/L 2-DG and 40 mmol/L DCA reduced L-lactate production substantially. On the other hand, metformin showed significant increase in L-lactate production (Fig 6A). For R848 treatment, effects of metabolic regulators on L-lactate production (120 ± 12%) match the results described for LPS with an additional significance for also the higher metformin concentration (Fig 6B). We also addressed the effect of these metabolic regulators on LPS and R848-mediated IL6 production (Fig 6C and 6D). Interestingly, with LPS present, 2-DG strongly reduced IL-6 production whereas DCA and metformin did not show significant changes in the production of IL-6 (Fig 6C). In R848-costimulated hPBMCs, 2-DG had similar effects compared to LPS stimulation. Furthermore, 10 mmol/L and 20 mmol/L metformin reduced IL-6 production in a significant manner (Fig 6D). These effects of the metabolic regulators 2-DG and metformin upon co-stimulation have also been investigated by other research groups [13, 14]. In summary, our results suggest that the custom assay is also able to detect metabolic changes in primary cells and that these metabolic changes can influence the immunostimulatory behavior of toll-like receptor (TLR) agonists as LPS and R848 for cytokine production such as IL-6.

## Discussion

Aberrant metabolic processes play an important role for many diseases such as cancer and immunological disorders and have therefore attracted the attention of various research fields [5–9]. In cancer, a metabolic reprogramming from aerobic to anaerobic glycolysis occurs to help the mutant cells fulfill their enormous energy need and gain building material for cell proliferation (Warburg effect) [5, 6]. The accumulation of L-lactate due to anaerobic glycolysis has for long not been seen as just metabolic waste, but is known to be a key molecule of tumor progression involved in angiogenesis, metastasis, immunosuppression and therapy resistance [6, 15].

The targeting of the metabolism by metabolic regulators like DCA, 2-DG and metformin has shown great therapeutic potential *in vitro* and *in vivo* as a combination with chemotherapy or radiation [16–19]. In order to get a deeper understanding of how the Warburg effect is achieved and how it can be influenced, it is necessary to observe the metabolic parameters namely being pH and L-lactate production. The custom L-lactate assay that we are proposing in this work can be used to study the significant changes of these parameters.

Certainly, attention for cell metabolism has also been rising for other research fields. Especially immune cells such as dendritic cells, macrophages and CD4^+^ or CD8^+^ T-cells upregulate their glycolysis level upon activation [7, 9, 20, 21]. Interestingly, immune cells that mostly utilize oxidative phosphorylation are considered anti-inflammatory while immune cells with higher levels of anaerobic glycolysis are comparably more of an inflammatory type [20, 21].

Although the existing research has already examined some connections of metabolism and physiological cell function, significantly more investigation is necessary to elucidate the complexity of this context. Unfortunately, commercially available kits for measurement of L-lactate as an indicator for metabolic status are expensive and not always applicable for the experimental setting due to its small range of measurement. On the contrary, the herein presented custom assay was shown to determine reliable, reproducible and precise measurement results while being cost-effective and suitable when tested for cancer and primary cells. The advantages of this approach are the reduced cost by more than 260-fold when compared to commercially available kits. Secondly, the capacity of the custom assay to measure higher L-lactate concentrations up to 12 mmol/L, which might be optimal for the investigation of high L-lactate producing cell lines. Additionally, this assay exhibits L-lactate concentration shifts as expected when treated with metabolic regulators being used in various research fields [9, 16–19].

Limitations of the assay are the LDH inhibitory activity of sodium pyruvate used in various culture media. However, this limitation could be overcome by raising the LDH enzyme concentration for the assay.

In conclusion, the custom assay we established clearly exhibits important advantages over the tested commercial kit, as it is highly cost-effective and presents an adequate working range for cells with low and high L-lactate production. We propose that the assay can be used in a variety of research fields, as the interest in metabolism increases for a better understanding of general cell function as well as for exploration of numerous therapeutic approaches.

## Supporting information

S1 File (ZIP)
